# Factors affecting reproductive traits in male snow leopards (*Unciauncia*)

**DOI:** 10.1530/RAF-20-0013

**Published:** 2020-11-11

**Authors:** Jason R Herrick, Cayla J Iske, Rachel M Santymire, Colleen Lynch, Mattina Alonge, Rebecca L Krisher, Cheryl L Morris

**Affiliations:** 1 Omaha’s Henry Doorly Zoo and Aquarium, Omaha, Nebrask, USA; 2 Colorado Center for Reproductive Medicine, Lone Tree, Colorado, USA; 3 Department of Animal Science, Iowa State University, Ames, Iowa, USA; 4 Davee Center for Epidemiology and Endocrinology, Lincoln Park Zoo, Chicago, Illinois, USA; 5 Riverbanks Zoo and Garden, Columbia, South Carolina, USA

**Keywords:** snow leopard, spermatozoa, nutrition, endocrinology

## Abstract

**Abstract:**

The population of snow leopards (*Unciauncia*) maintained in US zoos is no longer sustainable due to poor reproductive success. Our objective was to assess reproductive traits in male snow leopards and identify factors (markers of oxidative stress in seminal fluid, surveys of husbandry practices, gonadal and adrenocortical activity, dietary intake of various nutrients, and genetics) that may affect ejaculate traits and subsequent fertility. Ejaculates (2.9 ± 0.2 mL) from 32 male snow leopards (9.8 ± 0.7 years; 38.6 ± 0.8 kg) housed at 27 institutions contained 119.2 + 26.0 x 10^6^ spermatozoa, of which 75.1 ± 2.3% were motile and 28.6 ± 2.6% exhibited normal morphology. Overall, 34% of males produced <5 million spermatozoa and 27% of males produced spermatozoa with <20% normal morphology. Activity of superoxide dismutase (SOD) in the seminal fluid was negatively correlated (*P* < 0.05, *r*^2^ = 0.90) with normal sperm morphology. Husbandry practices, mean concentrations of fecal androgen metabolites (fAM), and baseline concentrations of fecal glucocorticoid metabolites (fGM), inbreeding coefficients, and generations each male was removed from the founders in their lineages were not correlated (*P* > 0.05) with the total number of spermatozoa or the proportion of spermatozoa with normal morphology. Total sperm count was positively correlated (*P* < 0.05, *R*^2^ = 0.86) with the weekly intake of polyunsaturated fatty acids (PUFA) and the proportion of spermatozoa with normal morphology tended (*P* < 0.10, *R*^2^ = 0.31) to be positively correlated with copper intake. Altering the nutrient composition of snow leopard diets could provide managers with a possible method of improving reproductive traits in this endangered species.

**Lay summary:**

The population of snow leopards (*Uncia uncia*) maintained in US zoos has been declining since 1993 due to poor breeding success. Our objective was to assess the reproductive traits of male snow leopards and identify factors (e.g. hormones, diet, genetics) that may be affecting the quality of semen produced and therefore subsequent fertility. Within a cohort of 32 male snow leopards maintained at 27 US zoos, we found that 34% produced less than 5 million sperm and 27% of males produced sperm where less than 20% looked normal. The quantity and quality of the recovered sperm was not correlated with husbandry practices, concentrations of hormones (androgens and glucocorticoids) in feces, or genetics. However, the number of sperm was positively correlated with polyunsaturated fatty acids in the diet. Altering the nutrient composition of snow leopard diets could provide managers with a possible method of improving reproductive traits in this endangered species.

## Introduction

The snow leopard (*Unciauncia*) was listed as an endangered species by the International Union for Conservation of Nature and Natural Resources in 1986 (McCarthy *et al.* 2017). Although recently upgraded to vulnerable, wild populations continue to decline with less than 3,400 mature individuals thought to exist (McCarthy *et al.* 2017). A number of *in situ* conservation efforts have been initiated to prevent this species from becoming extinct, ranging from studies of the species’ natural history to educating and supporting local communities (Jackson *et al.* 2010). The Association of Zoos and Aquariums’ (AZA) Species Survival Plan® (SSP) also participates in conservation efforts for this species by managing *ex situ* population of snow leopards that could be used to supplement or re-establish wild populations in the future. In addition, zoo populations of snow leopards are a valuable resource to study their basic biology and educate zoo visitors who will likely never have the opportunity to view a snow leopard in the wild.

The goal of the Snow Leopard SSP is to maintain a population of 150 individuals with at least 90% genetic diversity for 100 years (Soulé *et al.* 1986). Snow leopards have historically reproduced well in zoos, with steady population growth between the 1970s and the early 1990s (Tetzloff *et al.* 2019). However, the population has been declining since 1993 and is no longer considered sustainable. If current population trends continue, genetic diversity will fall below 90% in only 52 years. One of the primary factors contributing to these dire population projections is poor reproductive success. Since 2008, 40–49 pairs have been recommended to breed each year, but only 6–14 pairs per year have produced litters, resulting in average breeding success of only 19.3% per year. It is imperative that reproductive success improves in order for the zoo population to remain viable and contribute to the conservation of this species.

Despite the popularity of snow leopards in zoos, surprisingly little is known about their reproductive biology. There have only been three studies concerning the reproductive biology of male snow leopards (Johnston *et al.* 1994, Roth *et al.* 1994, 1996). In these studies, spermatozoa were recovered from all males (>31 million per ejaculate) and the proportion of spermatozoa with normal morphology was high (~60%). However, all three of these studies were conducted prior to 1997, when reproductive success was high and the population was growing. There have been no published reports on the reproductive biology of male snow leopards in over 20 years.

The current study was designed to characterize ejaculates from male snow leopards in the current zoo population and determine if reproductive traits were correlated to husbandry practices, androgen concentrations, adrenocortical activity, nutrition, or genetics. Our specific objectives were to: (1) collect and analyze ejaculate traits in snow leopards; (2) assess husbandry practices at each institution; (3) validate the use of fecal glucocorticoid metabolites (fGM) to evaluate stress physiology using an adrenocorticotropin hormone (ACTH) challenge; (4) monitor fecal androgen metabolite (fAM) concentrations throughout the breeding season; (5) determine concentrations of nutrients, minerals, and vitamins in snow leopard diets; and (6) identify correlations between reproductive parameters, husbandry practices, endocrine traits, nutrition, and genetics.

## Materials and methods

### Animals

Male snow leopards in this study were included in the Snow Leopard SSP and maintained in USDA and/or AZA-accredited facilities. All procedures were reviewed and approved by the Animal Care and Use Committees of each institution.

### Reproductive exams and semen collection

Between March 2009 and March 2019, 32 male snow leopards housed at 27 zoological institutions underwent reproductive exams and semen collections. According to studbook records, males between 2 and 19 years of age have sired offspring (Tetzloff *et al.* 2019). All males in the study fell within this age range. All males were immobilized using a combination of ketamine (5.3 ± 2.0 mg/kg, range: 2.0–12.4) and medetomidine or dexmedetomidine (0.06 ± 0.01 mg/kg, range: 0.01–0.39). This combination was further supplemented with midazolam (0.12 ± 0.02 mg/kg; *n* = 16 collections) or butorphanol (0.33 ± 0.08 mg/kg; *n* = 6 collections) at the discretion of the attending veterinarian at each institution. In some cases, isoflurane (*n* = 17 collections) or propofol (*n* = 8 collections) were used to increase the depth and/or duration of anesthesia. All immobilizations occurred during the breeding season (January to June) (Johnston *et al.* 1994, Roth *et al.* 1997).

Prior to semen collections, the prepuce and glans penis were visually inspected and the length and width of each testicle were measured with calipers to the nearest millimeter in order to calculate testicular volume (Howard 1993). Semen was collected via electroejaculation, using a protocol proven to be safe and effective for felids, including snow leopards (Johnston *et al.* 1994, Roth *et al.* 1996). Briefly, a lubricated probe (2.5 cm diameter) with three longitudinal electrodes was inserted into the rectum and used to apply a series of controlled stimuli (three sets of 30 stimulations) with increasing voltage (2–6 V per stimuli) to the accessory glands through the ventral wall of the rectum. At the conclusion of each series, the volume of recovered sample was determined and small aliquots (~5 µL) were used to evaluate pH (pH Indicator Strip; Millipore Sigma), the proportion of motile sperm (0–100%), and sperm morphology (fixed in 0.4% glutaraldehyde) (Howard 1993, Herrick *et al.* 2010*a*). The remaining sample was diluted 1:1 with HEPES-buffered Feline Optimized Culture Medium (FOCMH) (Herrick *et al.* 2010*a*, Herrick 2019) and maintained at room temperature. At the conclusion of the procedure, the samples recovered from each series were combined to determine the overall concentration of spermatozoa (x10^6^/mL) using a hemocytometer and the total number of recovered spermatozoa (x10^6^) was calculated. The combined sample was centrifuged (300 ***g*** for 10 min) and the supernatant was centrifuged again at 1100 ***g*** for 10 min. The final supernatant was aspirated and stored at −80°C for seminal fluid analysis and spermatozoa were cryopreserved (Crosier *et al.* 2006, Herrick *et al.* 2010*b*). The morphology of 200 spermatozoa per ejaculate were visually assessed at 1000x to determine the proportion of spermatozoa with normal morphology, as well as the proportions of spermatozoa with an abnormal head, midpiece, or tail according to previously published standards for feline spermatozoa that have been used to characterize individual males, or species, as normospermic (>60% spermatozoa with normal morphology) or teratospermic (<40% spermatozoa with normal morphology) (Howard *et al.* 1990, Howard 1993, Pukazhenthi *et al.* 2006*a*).

### Analysis of seminal fluid for oxidative stress markers

Samples of diluted (1:1 with FOCMH) seminal fluid (*n* = 9 males) were analyzed for concentrations of thiobarbituric acid reactive substances (TBARS), protein carbonyls (PC), DNA/RNA damage, superoxide dismutase (SOD), and glutathione peroxidase (GPx) using commercially available assay kits purchased from Cayman Chemical Company according to the recommendations of the manufacturer. Ferric reducing antioxidant power (FRAP) was also assessed as an indicator of total antioxidant capacity based on the reduction of ferric iron (Fe^+3^) to ferrous iron (Fe^+2^) by the colorimetric reaction of ferrous-tripyridyltriazine complex (Benzie & Strain 1996).

### Survey of husbandry practices

While visiting each zoo for the reproductive exams on 14 males, the cat’s exhibit and holding areas were observed and relevant animal care staff were interviewed to determine light exposure, relative proximity to other large carnivores, feeding practices, enrichment practices, and the number of care staff that interact with that individual.

### Validation of fecal glucocorticoid metabolite analysis using ACTH challenge

Fecal samples were collected daily 4 days before and after an injection of ACTH (Sigma A0298, Adrenocorticotropic Hormone, fragment 1–24 human and rat; 400 IU total) (Wielebnowski *et al.* 2002*a*, Young *et al.* 2004) from one male located at Omaha’s Henry Doorly Zoo and Aquarium (Omaha, NE). The administration of ACTH is intended to induce the secretion of cortisol from the adrenal glands, which will then be excreted in the feces within 96 h, as observed in cheetahs (Terio *et al.* 1999).

### Analysis of fecal concentrations of androgen and glucocorticoid metabolites

Fecal samples were collected three times per week during routine, daily maintenance of the animal’s exhibit and holding areas throughout the same breeding season as the semen collections (*n* = 14 males). Samples were stored at −20°C and shipped to the Lincoln Park Zoo Endocrinology Laboratory for analysis. For hormone extraction, 0.5 g of wet feces and 5 mL of 90% ethanol were mixed overnight, centrifuged (500 ***g*** for 20 min), and the supernatant poured into clean tubes (Reichert-Stewart *et al.* 2014).

We determined the fGM concentrations using a cortisol double antibody enzyme immunoassay (EIA) modified from Schell *et al.* (2017). First, 96 wells plates were coated with a goat anti-rabbit (GAR) antibody (1:1000, Arbor Assay, Ann Arbor, MI) and incubated at room temperature for 1 day. Contents were poured out, plates were blotted dry and a blocking buffer (Arbor Assay) was added. Blocked plates were incubated at room temperature for 1 day. The blocking buffer was poured off, plates were blotted dry and stored at 5°C until used. Fecal extracts were diluted from 1:20 to 1:80. On the day of the EIA analysis, polyclonal cortisol antiserum (R4866) and horseradish peroxidase (HRP) ligands (provided by C. Munro; University of California, Davis, California) were used at dilutions of 1:375,000 and 1:200,000, respectively. The cross-reactivities for the cortisol antiserum were cortisol, 100%, prednisolone, 9.9%; prednisone, 6.3%; cortisone, 5%; corticosterone, 0.7%; deoxycorticosterone, 0.3%; 21-deoxycortisone, 0.5%; 11-deoxycortisol, 0.2%; progesterone, 0.2%; 17α-hydroxyprogesterone, 0.2%; pregnenolone, 17α-hydroxypregnenolone, anderostenedione, testosterone, androsterone, dehydroepiandrosterone, dehydroisoandrosterone-3-sulfate, aldosterone, estradiol-17β, estrone, estriol, spironolactone and cholesterol, 0.1% (Young *et al.* 2004, Loeding *et al.* 2011). The fGMs were biochemically validated by determining: (1) parallelism between binding inhibition curves of dilutions of fecal extracts and the cortisol standards (*r* = 0.99); and (2) significant recovery of exogenous cortisol (3.9–1000 pg/well) added to fecal extracts (y = 0.960x + 3.402; *R*² = 0.998, *P* < 0.001). The intra- and inter-assay CV were <10 and <15% (*n* = 35 assays), respectively.

For fAM, a single antibody EIA system was used with testosterone horseradish peroxidase (HRP) ligands and polyclonal antiserum (R156/7; University of California, Davis, CA) at 1:30,000 and 1:10,000, respectively. Antiserum cross-reactivities for the testosterone antiserum were: testosterone, 100%; 5a-dihydro-testosterone, 57.37%; androstenedione, 0.27%; androsteron and DHEA, 0.4%; and cholesterol, 0.03%. Cross-reactivity was 0.02% for the following: β-estradiol, progesterone, pregnenolone, hydrocortisone, cholic acid, chenodeoxycholic acid, cholic acid methyl ester, dehydrocholic acid, deoxycholic acid, lithocholic acid, glycholic acid, taurodeoxycholic acid, taurocholic acid, taurochendeoxycholic acid, and glycochenodeoxycholic acid (Santymire & Armstrong 2010). The fAMs were biochemically validated for snow leopards by determining: (1) parallelism between binding inhibition curves of dilutions of fecal extracts and the testosterone standards (*r* = 0.993); and (2) significant recovery of exogenous testosterone (2.3–600 pg/well) added to fecal extracts (y = 1.029x − 6.258, *R*² = 0.994, *P* < 0.001). The intra- and inter-assay CV were <10 and <15% (*n* = 45 assays), respectively. Baseline concentrations for cortisol were determined by an iterative process in which values that exceed the mean plus 2 s.d. were excluded. The average was re-calculated and the elimination process repeated until no values exceed the mean plus 2 s.d. Baseline values were determined after all high values have been excluded (Brown *et al.* 1996).

### Nutritional analysis of diet

A sample of the cat’s diet (*n* = 14 males) was collected on the same day as the electroejaculation and stored at −20°C until analysis. Diets were consistent for at least 2 months prior to sample collection. Approximately 200 g of each diet sample was subsampled, dried at 55°C, ground in a commercial blender, and analyzed for chemical composition in the nutrition lab at Omaha’s Henry Doorly Zoo and Aquarium (Iske *et al.* 2016). Fatty acid profiles of raw meat diets were determined by gas chromatography at Iowa State University (Richter *et al.* 2012). Peak identification and quantification (Kramer *et al.* 2008) were determined on esterified lipid samples (Christie 1972) extracted from each diet sample (Folch *et al.* 1957). Mineral analyses of raw meat diets were conducted at Midwest Laboratories (Omaha, NE) according to Method 985.01 (Horowitz & Latimer 2006). Diet subsamples from each institution were sent to Arizona State University for analysis of vitamin A (retinol) and E (α-tocopherol) via reverse phase HPLC, as previously described (McGraw *et al.* 2006, Dierenfeld *et al.* 2009).

Weekly dietary intakes were calculated by multiplying the amount of diet fed by the number of days cats were fed per week, in order to account for variation in the number of fasting days. Weekly intakes were then multiplied by dry matter (DM) concentration of the diet to give weekly dry matter intake (DMI), which were then multiplied by nutrient content (%) to yield weekly intakes of each nutrient on a dry matter basis (DMB). Whole prey items, defined as non-living, whole animal carcasses, included rabbit, rat, fish, guinea pig, chicken, and quail, were not analyzed for chemical composition because the frequency of feeding and size of whole prey items indicated these items accounted for less than 10% of each animal’s diet.

### Population genetics

Pedigree information for all snow leopards in US zoos dating back to 1903, including all of the males in this study, is maintained by the AZA in a regional studbook (Tupa 2019). This data set was analyzed with population management software (Ballou *et al.* 2011, Faust *et al.* 2012) to determine (1) the inbreeding coefficient (F) for each male based on the relatedness of their dam and sire and (2) the number of generations between each male and the population’s founder animals from which they are descended (averaged for the maternal and paternal lineages).

### Statistical analysis

Multiple regression analysis was conducted using SAS® (SAS Inst. Inc. Cary, NC) with total sperm count and percent normal sperm morphology as the dependent variables and weekly dietary intake of macronutrients, fatty acids, vitamin A, and minerals, as well as seminal fluid measures of oxidative stress, as independent variables. Final statistical models only included significant variables. Age, body weight, frequency of feeding whole prey items, the number of fasting days, and genetic parameters for each animal were also analyzed via regression analyses.

Concentrations of fAM and fGM were log-transformed and analyzed using a linear mixed-effects model (including fixed and random effects) in R Core Team (2018) and R Studio Team (2018) using the REML method, assuming no within-group correlations in the *nlme* package (Pinheiro *et al.* 2018). Month was included as a fixed factor and male was included as a random factor. We measured the goodness of fit of the models by examining the correlation between model predictions and the observed data. We then examined the effects over time (across months) for the treatment identified as important from these models using Kruskall–Wallis ANOVA on Ranks with the Dunn’s method for multiple comparisons using Sigma Stat (version 11.0; Systat Software, Inc., Chicago, IL).

The Mixed Model procedure of SAS was used to evaluate the fixed effects of diet type (single or multiple diet types fed), frequency that cats were fed whole prey items (less than once per week, once per week, more than once per week), age (3–6, 7–10, and 11–16 years), and body weight (30–33, 35–41, and 42–49 kg) on sperm quality using ANOVA. Least squared means were used for pairwise comparisons.

For all analyses, *P* < 0.05 was considered indicative of a significant difference and *P* < 0.1 was assumed to indicate a statistical trend. All means are presented ± s.e.m.

## Results

### Semen collections

During a ten-year period between March 2009 and March 2019, reproductive exams and semen collections (*n* = 41) were performed on 32 male snow leopards (9.8 ± 0.7 years; 38.6 ± 0.8 kg) housed at 27 institutions (Table 1). The majority of males (23 of 32) were examined once, eight males were examined twice, and one male was examined three times, with 6 months to 4 years between collections. When multiple collections were performed on the same male, the mean values for all collections from that male were used for further analysis. Two males were unilaterally cryptorchid, but no other gross anatomical abnormalities were observed. Ejaculates (2.9 ± 0.2 mL) contained 119.2 ± 26.0 x 10^6^ spermatozoa (range 0 to 663.0 x 10^6^), of which 75.1 ± 2.3% were motile and 28.6 ± 2.6% exhibited normal morphology (Table 1). The most common abnormalities observed were spermatozoa with a proximal cytoplasmic droplet (19.9 ± 1.9%) and spermatozoa with a bent midpiece and a retained cytoplasmic droplet (19.4 ± 2.3%) (Table 1). Overall, 34.4% of evaluated males produced less than 5 x 10^6^ sperm and 27.3% of males had ejaculates in which less than 20% of the spermatozoa exhibited normal morphology.

**Table 1:** Results of reproductive examinations and semen collections performed on male snow leopards (*n* = 32 males) between 2009 and 2019.

	Mean (±s.e.m.)	Range
Age at collection (years)	9.8 ± 0.7	3–18
Body weight (kg)	38.6 ± 0.8	30.5–48.9
Total testicular volume (cm^3^)	18.5 ± 0.8	8.6–26.6
Ejaculate volume (mL)	2.9 ± 0.2	0.8–5.5
Ph	8.6 ± 0.05	7.7–9.0
Total sperm count (x10^6^)	119.2 ± 26.0	0.0–663.0
%Motile	75.1 ± 2.3	40.0–90.0
%Morphologically normal	28.6 ± 2.6	6.0–48.5
%Morphologically abnormal		
Proximal cytoplasmic droplet	19.9 ± 1.9	0.5–33.8
Distal cytoplasmic droplet	5.3 ± 1.1	0.0–21.0
Bent midpiece	8.7 ± 1.0	1.5–19.0
Bent midpiece w/retained cytoplasmic droplet	19.4 ± 2.3	1.5–45.5
Bent tail	9.0 ± 1.2	0.0–22.0
Tightly coiled tail	7.0 ± 3.7	0.0–81.5
Seminal fluid oxidative stress markers^1^		
Superoxide dismutase (U/mL)	3.3 ± 0.8	0.0–6.2
DNA/RNA damage (ng/mL)	27.4 ± 4.2	9.3–47.4
TBARS (µM)^2^	8.7 ± 0.8	6.5–12.7
Protein carbonyls (µM)	1.9 ± 0.6	0.0–5.5
FRAP (µM)^3^	165.3 ± 19.7	101.8–256.0

^1^*n* = 9 males examined during the 2016 breeding season using a 1:1 dilution of seminal fluid with culture medium; ^2^Thiobarbituric acid reactive substances; ^3^Ferric reducing antioxidant power.

Age (*R*^2^ = 0.16, *P* < 0.02) and testicular volume (*R*^2^ = 0.26, *P* < 0.05) were weakly, but significantly, correlated with sperm number, with more sperm being recovered from younger males and/or males with greater testicular volume. When grouped by weight, cats weighing 35–41 kg tended (*P* < 0.10) to have a higher proportion of normal spermatozoa (33.6%) than heavier cats (42–49 kg, 17.1%). Age and weight were not significantly correlated (*R*^2^ = 0.061, *P* > 0.1). Neither testicular volume nor ejaculate volume (*P* > 0.2) were correlated with sperm morphology. The activity of SOD (*R*^2^ = 0.90) in seminal fluid samples (*n* = 9 males) was negatively correlated (*P* < 0.05) with sperm morphology. None of the other markers of oxidative stress were correlated with ejaculate traits.

### Husbandry

Male snow leopards were cared for by two to seven people (mean = 5.3) (Table 2). All males were exposed to seasonal fluctuations in photoperiod and housed in large exhibits (approximate area, range 600–5000 ft^2^) with at least 12 ft of vertical space (Table 2). All but one male was part of a regular behavioral training program (Table 2). Males received enrichment at least three times per week and 12 of 14 males were provided with enrichment on a daily basis (Table 2). Animal care staff classified their male’s personality as calm in 12 of 14 cases and none of the males were considered aggressive by their caretakers (Table 2). None of the males were permanently housed in an enclosure adjacent to another large carnivore.

**Table 2:** Questions asked of animal care staff regarding male snow leopard’s (*n* = 14) environment and relevant husbandry practices.

Survey Question	Results		
	Answers, *n*	Mean ± s.e.m.	Range
How many keepers work with the male?		5.3 ± 0.5	2–7
Approximate dimensions of the cat’s holding and exhibit enclosures?		249.6 ± 60.4 m^2^	55.7–839.8
Is the cat exposed to seasonal changes in photoperiod and/or temperature?	Yes, 14 of 14		
While on exhibit, does the male have the option of remaining hidden from the public?	Yes, 14 of 14		
Is the cat fed one or more commercial diet(s) with occasional whole prey items?	Yes, 14 of 14		
If given whole prey items, how often (days/month)?		3.6 ± 0.8	1–12
How often does the male receive enrichment items?			
Daily	12 of 14		
≥3x per week	2 of 14		
Is the male part of an active behavioral training program?	Yes, 13 of 14		
In your opinion, would you characterize the cat’s temperament			
Calm	12 of 14		
Unpredictable	1 of 14		
Nervous	1 of 14		

### ACTH challenge

**Figure 1:**
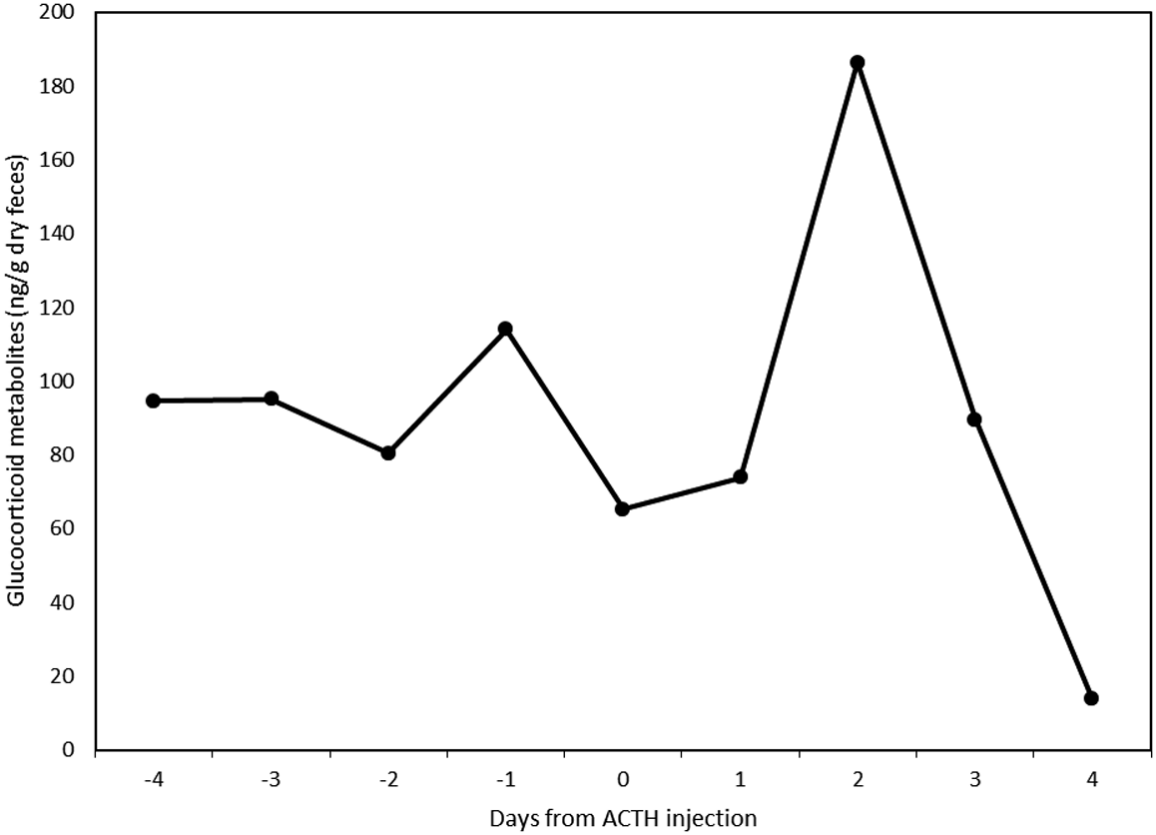
Fecal concentrations (ng/g dry feces) of glucocorticoid metabolites (fGM) in samples collected from one male snow leopard after an injection of adrenocorticotropin hormone (ACTH).

Mean fGMs before the ACTH were 89.8 ± 8.1 ng/g dry feces. FGMs (186.4 ng/g dry feces) increased on Day 2 after the ACTH injection (Fig. 1). This was a two-fold increase over mean fGM before injection. FGMs declined after Day 2 back to pre-injection values (90.9 ± 35.8 ng/g dry feces).

### Endocrine analysis

**Figure 2:**
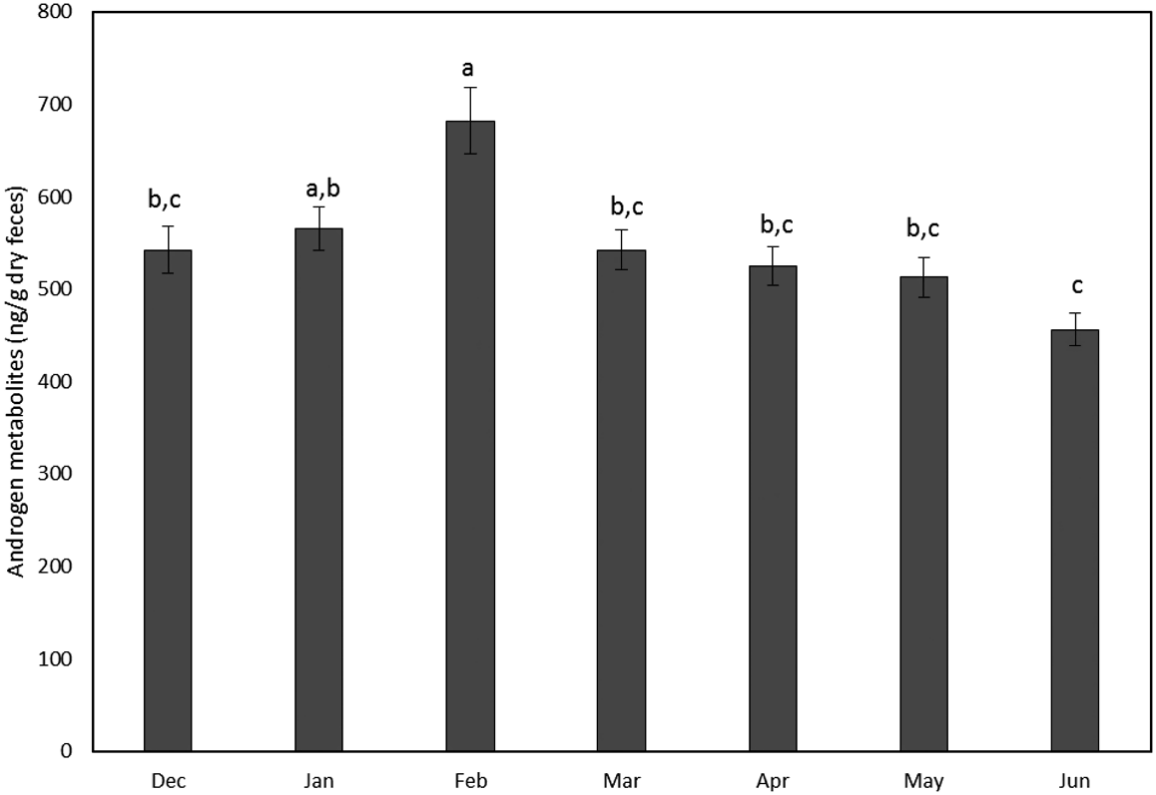
Fecal concentrations (ng/g; mean ± s.e.m.) of androgen metabolites (fAM) in samples collected from 14 male snow leopards during each month of the breeding season. Different letters (a, b, c) indicate a significant difference (*P* < 0.05) among months.

**Figure 3:**
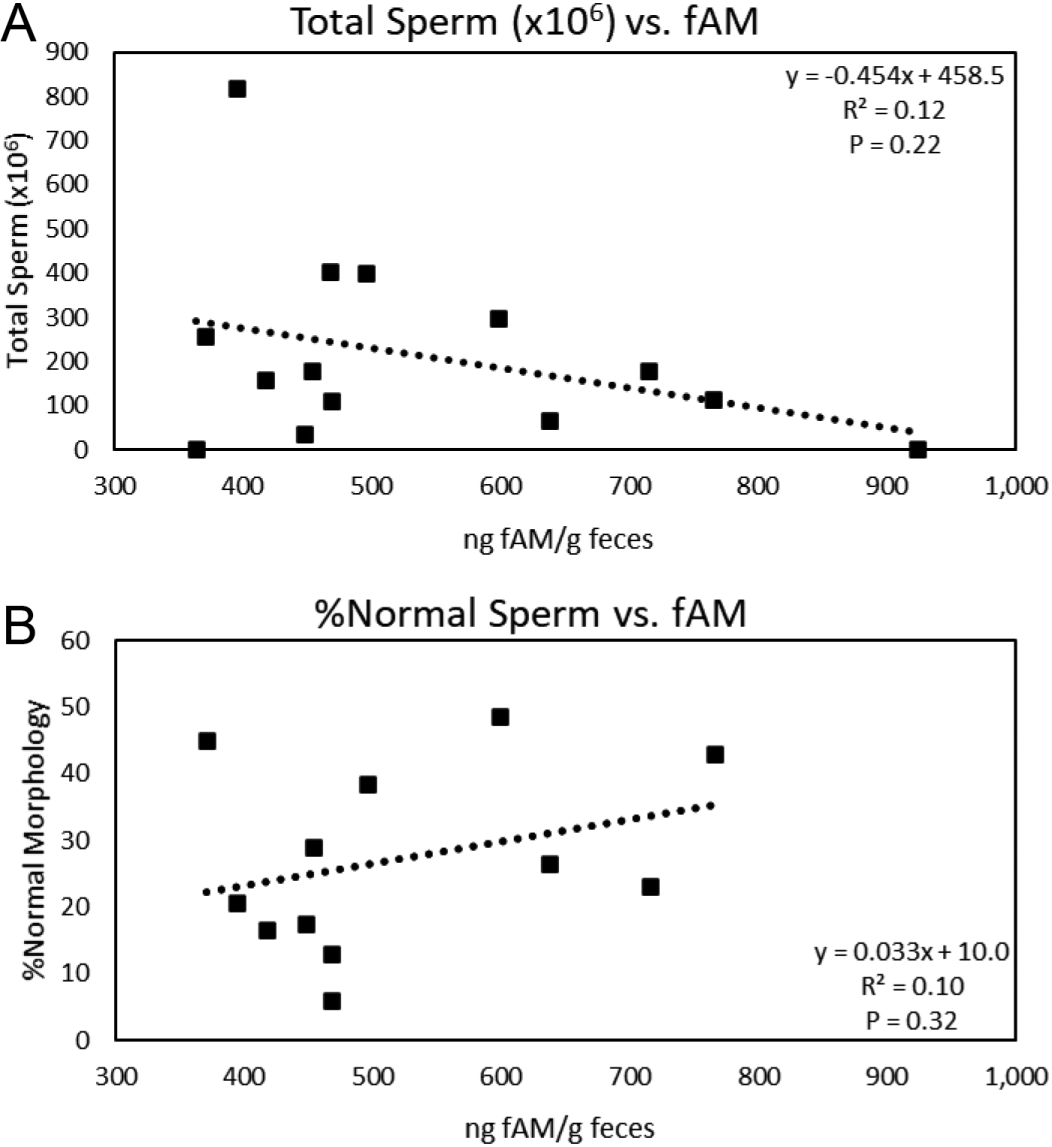
Linear regression analysis of the correlation between (A) the total number of spermatozoa (x10^6^) per ejaculate and (B) the proportion of spermatozoa with normal morphology and the mean concentration (ng/g) of fecal androgen metabolites (fAM) in samples collected throughout the same breeding season as the semen collection (*n* = 14 males).

**Figure 4:**
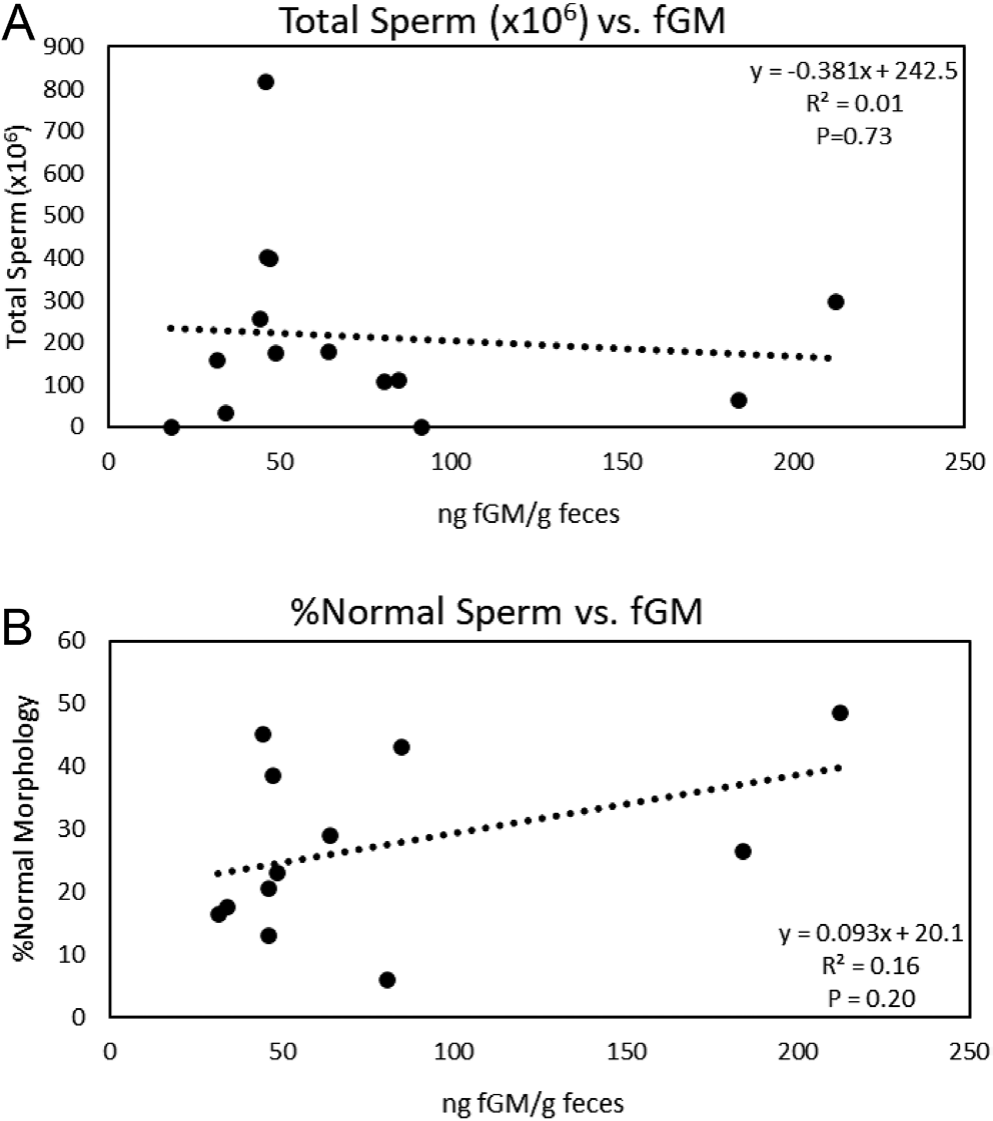
Linear regression analysis of the correlation between (A) the total number of spermatozoa (x10^6^) per ejaculate and (B) the proportion of spermatozoa with normal morphology and the mean baseline concentration (ng/g) of fecal glucocorticoid metabolites (fGM) in samples collected throughout the same breeding season as the semen collection (*n* = 14 males).

Concentrations of fAM ranged from 369.2 ± 33.9 to 992.5 ± 114.5 ng/g dry feces and varied (H_6_ = 39.72, *P* < 0.001) across months. Specifically, fAM peaked (*P* < 0.05) in February with the lowest concentrations in June (Fig. 2). Variation among males explained 28.3% of the variation in fAM concentrations not explained by month. Concentrations of fGM ranged from 55.1 ± 1.9 to 270.4 ± 17.1 ng/g dry feces across the males, which accounted for 48.3% of the variation not explained by month. Concentrations were different (H_6_ = 21.88, *P* = 0.001) across the months with January (114. 0 ± 5.8 ng/g dry feces) higher (*P* < 0.05) than April (110.1 ± 7.7 ng/g dry feces) and May (104.6 ± 7.1 ng/g dry feces), but all other months were similar (*P* < 0.05) (mean, 111.1 ± 4.3; range, 97.0–114.2 ng/g dry feces). Neither fAM or fGM were correlated (*P* ≥ 0.2, *R*^2^ ≤ 0.16) with the total number of spermatozoa collected or the proportion of spermatozoa with normal morphology (Figs 3 and 4).

### Nutrition

All cats were fed raw, commercially manufactured beef, horse, or pork-based diets (900–1800 g as fed; 300–600 g DMB) from Nebraska Brand^®^ (North Platte, NE), Triple A Brand Meat Company^©^ (Burlington, CO), Milliken Meat Products Ltd (Markham, Ontario), or Sustainable Swine Resources (Sheboygan Falls, WI) 4–7 days per week (0–3 fasting days/week). Whole prey items were provided 3.6 ± 0.8 days/month and accounted for less than 10% of the diet (by wet weight) due to the small size (e.g. rabbit, rat, fish, guinea pig, chicken, or quail) of these items. Cats that consumed a pork-based diet (*n* = 2) had more (*P* < 0.05) normal sperm (45.8%) compared to those fed only horse (26.6%) or beef (11.3%). Cats fed whole prey once per week produced ejaculates with a higher (*P* < 0.05) proportion of normal spermatozoa (37.2%) than cats fed whole prey less than once per week (14.6%), but were not different than cats fed whole prey more than once per week (27.5%). The number of times cats were fed whole prey items did not affect (*P* > 0.05) the total number of spermatozoa per ejaculate.

Weekly intakes of dry matter (2274.8 ± 186.2 g), organic matter (2089.0 ± 170.3 g), crude protein (1290.5 ± 107.4 g), total fat (728.9 ± 74.0 g), total dietary fiber (122.3 ± 11.6 g) and metabolizable energy (11,727.1 ± 1014.4 kcal) were not correlated (*P* > 0.05) with reproductive traits (data not shown). Total sperm count was positively correlated (*P* < 0.05, *R*^2^ = 0.86) with weekly intake of dietary polyunsaturated fatty acids (PUFA). Total sperm count also tended (*P* < 0.10) to be positively correlated with weekly intakes of dietary phosphorus (*R*^2^ = 0.27) and retinol (vitamin A, *R*^2^ = 0.23) and negatively correlated with weekly dietary intake of manganese (*R*^2^ = 0.21). The proportion of spermatozoa with normal morphology was negatively correlated (*P* < 0.05) with weekly intake of retinyl acetate (*R*^2^ = 0.38) and magnesium (*R*^2^ = 0.29) and tended (*P* < 0.10) to be negatively correlated with weekly dietary intake of margaric acid (*R*^2^ = 0.31) and positively correlated with weekly copper intake (*R*^2^ = 0.31) (Table 3).

**Table 3:** Dietary variables (based on calculated weekly intake) that were correlated (*P <* 0.10) with the total number of spermatozoa per ejaculate or the proportion of spermatozoa with normal morphology in male snow leopards (*n* = 14).

Ejaculate trait/weekly nutrient intake	*R*^2^	*P*-value	Association
Total sperm (x10^6^) per ejaculate			
Polyunsaturated fatty acids	0.86	0.0003	Positive
Phosphorus	0.27	0.06	Positive
Manganese	0.21	0.06	Negative
Retinol	0.23	0.08	Positive
Proportion of spermatozoa with normal morphology			
Retinyl acetate	0.38	0.03	Negative
Magnesium	0.29	0.04	Negative
Margaric acid	0.31	0.06	Negative
Copper	0.31	0.07	Positive

A further investigation of the ingredient lists provided by the manufacturers indicated that two of the commercial diets (http://www.nebraskabrand.com/docs/ClassicSheet2019pdf.pdf; http://www.nebraskabrand.com/docs/beefsheet2019pdf.pdf) contained soybean meal, which is known to contain bio-active phytoestrogens (Cederroth *et al.* 2010). Males fed these diets had reduced (*P* < 0.05) testicular volume (17.9 ± 1.6 cm^3^) compared to males fed diets without added soy products (23.8 ± 1.4 cm^3^), but the total number of sperm per ejaculate and the proportion of normal sperm were not different (*P* > 0.05). Diets with added soy products did not affect (*P* > 0.05) hormone concentrations (fAM and fGM), but there was a significant (*P* < 0.05) interaction between diet and month for both fAM and fGM (data not shown). Within each month, fAMs were not affected (*P* > 0.05) by diet. For males fed diets with added soy, fAM peaked (*P* < 0.05) in February, but were similar (*P* > 0.05) across other months. For males fed diets without added soy, fAMs were higher (*P* < 0.05) in February and January than in April and June. For fGMs, males on diets with added soy had higher (*P* < 0.05) fGM concentrations in February, March, April and June than males fed diets without added soy.

### Population genetics

The 32 males in the study were 4.44 ± 0.22 generations descended from founders, including one wild-born individual (0 generations). The mean inbreeding coefficient was 0.018 ± 0.003, with all values (range, 0–0.052) falling below the estimated value for the offspring of first cousins (*F* = 0.0625). Neither parameter was correlated (*R*^2^ < 0.1, *P* > 0.2) with the total number of spermatozoa per ejaculate or the proportion of normal spermatozoa (data not shown).

## Discussion

Snow leopards have been maintained in US zoos for over 100 years, with successful breeding programs starting in the 1950s and steady population growth until the early 1990s (Wharton & Mainka 1997). Breeding success has declined in recent years and the sustainability of the zoo-based population is uncertain. The current study was initiated to improve our understanding of reproduction in male snow leopards and identify factors that could be linked to recent declines in reproductive success. In a cohort of 32 male snow leopards, we found that 34% of males produced <5 million spermatozoa and 27% of males produced spermatozoa with <20% normal morphology. Of the numerous endocrine, environmental, and genetic factors evaluated, weekly intake of polyunsaturated fatty acids was the only factor that was highly correlated with reproductive traits.

Ejaculate traits, including the quantity and quality of recovered spermatozoa, are known to vary widely between felid species (Howard 1993, Pukazhenthi *et al.* 2006*b*). For example, tigers (*Pantheratigris*) and ocelots (*Leoparduspardalis*) produce large quantities of spermatozoa with >60% normal morphology, while the proportion of normal spermatozoa in the ejaculates of cheetahs (*Acinonyx jubatus*) and clouded leopards (*Neofelisnebulosa*) are <20% (Byers *et al.* 1990, Howard 1993, Swanson *et al.* 2003, Pukazhenthi *et al.* 2006*a*, Crosier *et al.* 2007). Results from our current study would place snow leopards near the middle of this range, along with the majority of other felid species (Brown *et al.* 1991, Howard 1993, Swanson *et al.* 2003, Thiangtum *et al.* 2006, Herrick *et al.* 2010*a*). What is most alarming about our findings is not the number of spermatozoa or the proportion of spermatozoa with normal morphology, but how different our results are compared to previous studies of male snow leopards conducted in the early 1990s. In the previous studies, the total number of recovered spermatozoa ranged from 31 to 373 x 10^6^ (Roth *et al.* 1996). The low end of this range (31 x 10^6^) is significantly higher than values for 34% of the males in our study (<5 x10^6^ spermatozoa), including seven males with aspermic (no spermatozoa) ejaculates. Differences in the proportion of normal spermatozoa per ejaculate were even more dramatic, with 27% of the males in the current study exhibiting <20% normal spermatozoa and none of the males in our study producing >50% normal spermatozoa, compared to values as high as 63% normal spermatozoa in previous studies (Roth *et al.* 1994).

Studbook records indicate that 23 of the males in our study (127.1 ± 24.3 million spermatozoa, 29.1 ± 3.3% normal morphology) have sired at least one offspring in their lifetime. However, comparisons between these proven males and those males that have not sired offspring (unproven) are complicated due to the limited records available for each male’s breeding history. Specifically, studbook records do not indicate if the male ever had access to a female, if that access occurred at times the female was receptive, if copulation actually occurred, and if the female was known to be fertile. For example, the male that produced the highest number of spermatozoa in this study has never sired offspring, but he has never been paired with a female. Even for the proven males, the ability to sire offspring earlier in their lives does not necessarily ensure fertility at the time of semen collection. As a result, we were not able to correlate ejaculate traits and fertility. However, studies of artificial insemination in domestic cats indicate that pregnancy can only be achieved using <10 million spermatozoa if those spermatozoa are deposited directly into the uterine horns or oviduct (Tsutsui *et al.* 2000, Swanson 2019). Similarly, extensive research comparing spermatozoa from normospermic (>60% normal) and teratospermic (<40% normal) domestic cats have indicated that a low incidence of spermatozoa with normal morphology is associated with compromised sperm function, including capactitation, zona pellucida penetration, and oocyte activation (Howard *et al.* 1991, Long *et al.* 1996, Pukazhenthi *et al.* 1996, Villaverde *et al.* 2013).

Since the previous studies of snow leopards were performed on different animals by other researchers, procedural differences must be considered. All collections reported by Johnston *et al.* (1994) and some (proportion not specified) of the collections in Roth *et al.* (1996) utilized ketamine and xylazine to immobilize the males, compared to ketamine and medetomidine in the current study. Xylazine and medetomidine are both agonists of alpha-2 adrenergic receptors, but medetomidine has a higher binding affinity for these receptors than does xylazine (Schwartz & Clark 1998) and is more effective than xylazine for inducing the release of spermatozoa into the urethra (Swanson *et al.* 2017), suggesting the use of medetomidine in the current study was not the cause of the reduced sperm recovery in the present study. The remaining collections reported by Roth *et al.* (1994, 1996) utilized a combination of tiletamine and zolazepam. In other species, this combination of drugs allows the recovery of ejaculates with similar volume, sperm concentration, motility, and morphology as collections using ketamine and medetomidine (Barros *et al.* 2009, Santiago-Moreno *et al.* 2011). In the previous and current studies, semen was collected by eletroejaculation using a similar size probe (~2.5 cm), number of stimuli (80–90), and voltages (2–5 V) (Johnston *et al.* 1994, Roth *et al.* 1994, 1996). Finally, one of the co-authors of the Roth *et al.* studies (W.F. Swanson) trained J. Herrick on semen collection and evaluation in felids (Herrick *et al.* 2010*a*). For studies conducted by different personnel nearly 25 y apart, methodologies were remarkably similar, and we feel confident that differences in ejaculate traits are representative of the populations at the time of collection and not an artifact of procedural changes over time.

If the quantity and quality of spermatozoa produced by male snow leopards has indeed declined in the last 20–25 years, the obvious question is what factors have contributed to this change? Environmental factors can have a significant impact on reproductive success in felids (Mellen 1991, Wielebnowski *et al.* 2002*a*, Fazio *et al.* 2019). Although zoo exhibits and husbandry practices have improved in the last 20–25 years, with an ever-increasing emphasis on animal welfare, we do not always know how these changes affect reproduction. In the case of seasonally breeding species like snow leopards, exposure to an inappropriate photoperiod can alter gonadal activity (Brown *et al.* 2002, Graham *et al.* 2004, Newell-Fugate *et al.* 2007). Other factors, such as exhibit design, proximity to predators, and the relationship between animals and their caretakers have also been correlated to reproductive success (Mellen 1991, Koester *et al.* 2015, Fazio *et al.* 2019). Significant variation between husbandry practices at participating zoos made it difficult to statistically analyze the effects of some of these factors. However, available space and exposure to natural changes in photoperiod, two factors expected to be highly correlated with reproductive function in snow leopards, were remarkably similar across institutions. All institutions provided cats with enrichment multiple times per week, with most animals receiving some type of daily enrichment. The majority of the males also participated in positive reinforcement training programs, which has been associated with successful breeding in fishing cats (*Prionailurusviverrinus*) (Fazio *et al.* 2019). Although we did not objectively evaluate the relationship between males and their caregivers or animal temperament, the majority of keepers in our study felt that they had a good relationship with their animal and characterized the animal’s temperament as calm.

Concentrations of fGM were also monitored throughout the breeding season (Graham & Brown 1996) to provide some indication of the animal’s perception of their environment and the amount of stress they are experiencing that may not have been apparent upon visual inspection of the enclosures by researchers (Carlstead *et al.* 1992, Carlstead *et al.* 1993, Wielebnowski *et al.* 2002*a*). The use of fGMs to evaluate snow leopard stress physiology was validated using an ACTH challenge, similar to observations in other felid species (Carlstead *et al.* 1992, Graham & Brown 1996, Terio *et al.* 1999, Wielebnowski *et al.* 2002*a*, Young *et al.* 2004). There was no correlation between fGM and either the total number of recovered spermatozoa or the proportion of normal sperm per ejaculate, similar to reports in male cheetahs (Koester *et al.* 2015). However, ejaculate traits in the male cheetahs and ovarian activity in female cheetahs can be negatively influenced by public exposure and an increased number of care givers even though correlations with fGM were not significant. (Wielebnowski *et al.* 2002*b*, Koester *et al.* 2015, Koester *et al.* 2017). As pointed out by Koester *et al.* (2015), these results do not indicate ejaculate traits are independent of stress, just that the mechanism(s) affecting ejaculate traits in response to environmental/psychological stress may not be mediated by glucocorticoid secretion (MacDougall-Shackleton *et al.* 2019).

Monitoring concentrations of fAM is an effective means of determining the steroidogenic activity of the testes, allowing for the establishment of normative values for each species (Morais *et al.* 2002, Herrick *et al.* 2010*a*, Fanson *et al.* 2012), and characterization of seasonal patterns of reproduction in felids (Brown *et al.* 2002, Fanson *et al.* 2010). However, the relationship between androgen production, reflected in either serum or fecal concentrations, and ejaculate quality has not been well established. Single studies have reported correlations between serum androgen concentrations and the total number of spermatozoa recovered from cheetahs (Wildt *et al.* 1993) and the proportion of normal spermatozoa in domestic cats (Howard *et al.* 1990), but these same relationships have not been consistent in other studies (Brown *et al.* 1991, Barone *et al.* 1994, Muller *et al.* 2012, Koester *et al.* 2015). In the present study, the variation observed in the quantity of recovered spermatozoa or the proportion of normal spermatozoa per ejaculate in snow leopards was not correlated to androgen production.

Although diets for captive felids have been formulated to meet or exceed the National Research Council’s (NRC) nutrient requirements for domestic cats , it is not always known how well these diets match the nutrient composition of the animal’s natural diet or if and how specific nutrient requirements differ between domestic and exotic species. Similarly, dietary insufficiencies of vitamins, minerals, taurine, linoleic acid, and arachidonic acid negatively affect reproduction in domestic cats (MacDonald *et al.* 1984, Sturman 1991, Fascetti *et al.* 2000, Swanson *et al.* 2003), yet variations in these nutrients among diets for zoo carnivores are not well defined. Therefore, nondomestic species in zoos may experience subtle nutritional deficiencies or excesses that can affect their fertility, without overt effects on the animal’s health (Allen & Ullrey 2004, Howard & Allen 2008). In the current study, dietary intake of various nutrients, including minerals (phosphorus and copper), fatty acids (PUFA), and retinol, were each positively correlated with sperm quantity or quality in male snow leopards, consistent with studies in other species (Huang & Hembree 1979, Rode *et al.* 1995, Kowal *et al.* 2010, Safarinejad 2011, Risso *et al.* 2016, 2017, Narasimhaiah *et al.* 2018, Santos *et al.* 2019).

A retrospective analysis of the ingredients of the diets fed to the snow leopards in our study indicated that two of the diets fed to six males were supplemented with soy protein by the manufacturer. Soy products are a common source of dietary isoflavones with phytoestrogen activity that have been associated with reproductive dysfunction in males (Cederroth *et al.* 2010). For example, recent studies have suggested that the reduced fertility of southern white rhinoceroses (*Ceratotheriumsimumsimum*) in zoos may be linked to the phytoestrogen content of their diets (Tubbs *et al.* 2016). The male snow leopards that were fed diets with added soy proteon had reduced testicular volume and altered patterns of androgen production during the breeding season, but no significant effects were seen on ejaculate traits or hormone concentrations. However, our analysis only took into consideration one potential source of phytoestrogen. The horses, cattle, and pigs, as well as rodents, rabbits, and birds, that made up the diets of the snow leopards were likely fed diets containing phytoestrogens prior to processing, which may have increased the content of phytoestrogens in the resulting meat (Gatta *et al.* 2013, Dal Bosco *et al.* 2015). Therefore, while the diets with the added soy protein likely contained more phytoestrogens than the other diets, none of the cats were fed a diet that can be assumed to be free of phytoestrogens and additional, controlled studies will be necessary to better understand these possible effects.

Male reproductive parameters can also be significantly affected by genetic diversity (Asa *et al.* 2007, Malo *et al.* 2010). In felids, male cheetahs, pumas, and lions from isolated populations with reduced genetic diversity have an increased incidence of spermatozoa with abnormal morphology (O’Brien *et al.* 1983, Brown *et al.* 1991, Roelke *et al.* 1993). In the absence of molecular data, we examined the correlation between pedigree derived inbreeding coefficients and ejaculate traits. However, inbreeding coefficients estimated from pedigree data are dependent on the assumption that the population’s founders were unrelated. If founder animals were related, pairings in each generation could have higher levels of inbreeding than expected, and more genetic diversity could be lost over time than inbreeding coefficients suggest (Ito *et al.* 2017). In these cases, the loss of diversity over time may be reflected in correlations with the number of generations an individual is removed from the founders in their lineages. Although studies of molecular genetics in snow leopards are necessary to definitively exclude an effect of genetics, the lack of significant relationships between both inbreeding coefficient and generations from founders and the reproductive traits of males in our study suggests a limited role for genetics.

The results of this study provide a comprehensive assessment of ejaculate traits, endocrinology, husbandry, nutrition, and genetics in male snow leopards. It appears that a significant number of males in the current, US zoo population have reduced quantities of spermatozoa and an increased proportion of spermatozoa with abnormal morphology, which differs from previous reports of this same population in the 1990s. Although our sample size was relatively small (32 males, 14 males used for regression analysis with husbandry factors), it includes the equivalent of 46% of the males in the US population (*n* = 69), with regression analysis performed on 20% of these males. Of the factors evaluated, nutrition appeared to be the primary factor associated with reproductive traits, but prospective studies in which reproductive traits are evaluated following specific changes in diet composition are needed to validate our findings. Such controlled studies are especially important because some of the nutrients that were positively associated with ejaculate traits, have been associated with negative effects on reproduction and/or health when provided in excess (Gosselin *et al.* 1988, Bechert *et al.* 2002, Tvrda *et al.* 2015). Comparative studies in other species are also warranted, since all felid species maintained in US zoos are fed essentially the same diets. If controlled, prospective studies verify a direct effect of nutrients on ejaculate traits, altering the composition of snow leopard diets could provide managers with a possible method of improving ejaculate traits in male snow leopards.

## Declaration of interest

The authors declare that there is no conflict of interest that could be perceived as prejudicing the impartiality of the research reported.

## Author contribution statement

J R H designed the experiments and performed all reproductive exams, semen analyses, and husbandry practice surveys. C J I performed or coordinated all nutritional analyses, conducted assays for markers of oxidative stress, and completed relevant statistical tests. R M S validated assays for fecal androgen and glucocorticoid metabolites, supervised the analysis of samples by M A, and performed statistical analyses of all endocrine data. R L K provided laboratory supplies and salary for J R H. C L M assisted with nutritional analyses and data interpretation. C L conducted studbook analysis and provided data on inbreeding coefficients and generations. All authors participated in manuscript preparation.
